# Feline foamy virus seroprevalence and demographic risk factors in stray domestic cat populations in Colorado, Southern California and Florida, USA

**DOI:** 10.1177/2055116919873736

**Published:** 2019-09-16

**Authors:** Sarah Kechejian, Nick Dannemiller, Simona Kraberger, Carmen Ledesma Feliciano, Martin Löchelt, Scott Carver, Sue VandeWoude

**Affiliations:** 1 Department of Microbiology, Immunology, and Pathology, College of Veterinary Medicine and Biomedical Sciences, Colorado State University, Fort Collins, CO, USA; 2 The Biodesign Center for Fundamental and Applied Microbiomics, Center for Evolution and Medicine, School of Life Sciences, Arizona State University, Tempe, AZ, USA; 3 Division of Infectious Diseases, University of Colorado Anschutz Medical Campus, Aurora, CO, USA; 4 Department of Molecular Diagnostics of Oncogenic Infections, Research Program Infection, Inflammation and Cancer, German Cancer Research Center (Deutsches Krebsforschungszentrum Heidelberg, DKFZ), Heidelberg, Germany; 5 School of Biological Sciences, University of Tasmania, Sandy Bay, Tasmania, Australia

**Keywords:** Feline foamy virus, retrovirus, United States, spumaretrovirinae, epidemiology

## Abstract

**Objectives:**

Our study aim was to document the seroprevalence and associated risk factors of feline foamy virus (FFV) infection in domestic cat populations presented to animal shelters located in Southern California, Colorado and Florida, USA.

**Methods:**

We used a glutathione S-transferase capture ELISA targeting the FFV Gag antigen to screen domestic cat serum collected from cats with unknown owners at eight different animal shelters from Colorado (n = 105, three shelters), Southern California (n = 172, three shelters) and Florida (n = 31, two shelters). χ^2^ statistics determined location effect on seroprevalence. Bayesian generalized linear models were used to explore age and sex as potential risk factors for infection.

**Results:**

FFV seroprevalence was 64.0% across all locations. Seroprevalence by location was as follows: Southern California 75.0%, Colorado 52.4% and Florida 41.9%, with Southern California’s seroprevalence being significantly higher. Age had a significant effect on model fit for all locations, with adults having a higher probability of being infected. In Colorado, sex also had a significant effect on model fit, with males having a higher probability of being infected.

**Conclusions and relevance:**

We have documented that FFV is extremely common in stray domestic cat populations across varied geographic and ecological niches throughout the USA. Adult cats are at a higher FFV infection risk than young cats. FFV has been associated with a higher risk of other retroviral infections and has been implicated in several chronic diseases of cats. Additional epidemiological and clinical studies are warranted to investigate the potential impacts of FFV on domestic cat health.

## Introduction

Feline foamy virus (FFV) is an RNA retrovirus of the subfamily *Spumaretrovirinae*.^1^ Foamy virus success, defined by having a relatively high prevalence globally, is, in part, due to lifelong persistent infections that do not result in defined pathologies,^2,3^ coupled with unique replication strategies and highly conserved viral genomes.^4^ FFV was described in 1969 because of its cytotoxicity as a cell culture contaminant, and despite being recognized as a global infection in cats, few studies have documented direct clinical effects.^1^ The observed apathogenicity of FFV is potentially due to active replication of the virus typically being restricted to the oral mucosa, with only latent infection occurring in other tissue types.^5^ The transmission of FFV through oral cavity shedding is thought to require direct contact between animals, either via amicable contacts or biting.^5,6^

While there is investigation of FFV as a vaccine and gene therapy vector,^7–10^ its potential associations with the occurrence of chronic kidney disease in domestic cats^10,11^ and its potential to exacerbate other retroviral infections, such as feline immunodeficiency virus (FIV) and feline leukemia virus (FeLV),^6,12–15^ indicate that this agent may be relevant to domestic cat health. Experimental inoculations via intramuscular and/or intravenous infections in domestic cats have failed to identify overt acute clinical disease, but histological abnormalities in kidney and lung tissue have been reported.^10,11^ FFV has also been associated with polyarthritis in male cats.^16^ Determining the prevalence and some basic risk factors for FFV infection is fundamental to contextualizing the virus’ current and potential effects on domestic cat populations.

To date, however, many questions about FFV ecology, prevalence and demographic risk factors have yet to be definitively answered. Studies in other countries (Australia, Taiwan, Japan, Germany) have indicated that prevalence – determined via multiple screening techniques – varies between 30% and 70% of sampled populations.^2,6,17–19^ There is little published data documenting the prevalence of FFV in domestic cat populations in the USA,^20^ since regular screening for FFV infection is not practiced. To address this knowledge gap, we retroactively screened stray domestic cat serum samples collected from individuals living in three areas (Colorado, Southern California and Florida) for anti-FFV antibodies to determine prevalence and understand the risk factors associated with infection in these populations.

## Materials and methods

### Study design

Eight different shelters, three in Colorado (Montrose Animal Shelter, Boulder Humane Society and Second Chance Humane Society), three in Southern California (Ventura County Animal Shelter, Corona Animal Shelter and Escondido Animal Shelter) and two in Florida (Veterinary Center and Feral Cat Coalition) participated in this study between 2009 and 2011, with procedures approved by the Colorado State University Institutional Animal Care and Use Committee prior to implementation. The sample archive used was initially assembled to assess common feline viral infections in free-ranging domestic cats across various states.^21^ Blood samples were collected from stray domestic cats either upon admission to shelters (non-owner surrender) or through trap–neuter–return programs in Colorado and Southern California from 2009–2011, and from Florida in 2011, as previously described.^21^ Samples were collected prior to housing in the shelter facility to avoid measuring shelter-acquired infections. Demographic data collected by shelter veterinarians and used in this study included age (adult or young as classified by shelter personnel, relating to pre- and post-sexual maturity), sex (male or female) and location (Southern California, Florida or Colorado). Detailed age estimates, breed and ownership data were not available.^21^

### ELISA

Sera were tested in duplicate on separate 96-well plates at 1:50 dilution using a non-quantitative GST capture ELISA targeting the FFV Gag antigen, as previously described.^10,22^ This validated assay was shown to have high specificity and a higher sensitivity for detection of FFV antibodies in naturally and experimentally infected cats than immunoblots.^22^ A positive result was defined as having an optical density absorbance over [2 × (meanGag + 3 SD)], with ‘meanGag’ being the average negative control absorbance and ‘SD’ being the standard deviation of the negative control absorbance. This cut-off calculation was revised for each run, assuring each analysis was compared with its own negative control. The procedure was repeated with an additional step of pre-incubating sera with GST antigen for samples that had an average absorbance near the cut-off value, or that had noticeably large differences in absorbance between duplicates.

Serum from an experimentally infected domestic cat was used as a positive control and serum from a specific pathogen-free (SPF) cat was used as the negative control.^10,19^ The positive control was infected via a 1 ml intramuscular injection and a 1 ml intravenous injection of FFV infected cells. The SPF cat colony was screened for FFV using PCR and ELISA analysis.^10^ All plates were run in the same laboratory by the same individual over the course of 1 month. Reagents were remade on an as-needed basis. ELISA plates were prepared with fresh coating buffer the night prior to use.

### Statistical risk factor analysis

FFV seroprevalence in each location (Southern California, Colorado and Florida), as well as across all locations, was calculated, stratified by sex (male and female) and age (young and adult). A χ^2^ test was used to compare FFV seroprevalence in the three locations, followed by post-hoc pairwise χ^2^ tests to determine how locations differed, with *P* values adjusted for inflation owing to multiple comparisons via Benjamini–Hochberg methods.^23^

We conducted a risk factor analysis on each location and across all locations to evaluate sex and age, and the interaction between sex and age, using Bayesian generalized linear models (GLMs; a style of linear regression that accounts for response variables with non-normal error distributions) with binomial error distributions.^24^ All predictor variable combinations and a null model were considered. For each coefficient (ie, variable combination), we used weakly informative priors and extracted a 95% credible interval from the posterior distribution. Any coefficient whose 95% credible interval did not contain 0 was considered significant. Bayesian GLMs were ranked and compared using an Akaike information criterion (AIC), an estimator of the relative quality of a statistical model when compared with other models for a given set of data. The model with lower AIC values was considered to better fit the data and subsequently better predict FFV infection. Models within two AIC units were considered indistinguishable (E Gagne, 2018, personal communication);^25^ therefore, if a model had a significant predictor and was within two AIC of the best fit model, it was considered to reveal the most important risk factor for FFV infection in stray domestic cats.

## Results

We analyzed 105 unique samples from Colorado, 172 samples from Southern California and 31 samples from Florida. Seroprevalence of FFV was high in all three locations, with an overall seroprevalence of 64.0% (95% confidence interval [CI] 58.2–69.3). The Southern California shelters had the highest seroprevalence (75%, 95% CI 67.8–81.3), followed by Colorado (52.4%, 95% CI 42.4–62.2) and Florida (41.9%, 95% CI 24.6–60.9). A significant association between FFV seroprevalence and location was found (χ^2^ = 21.725, *P* <0.001). Seroprevalence broken down by sex and age in each sampling location is displayed in Table 1. Post-hoc pairwise χ^2^ tests indicated that cat samples from Southern California were significantly more likely to be positive than Colorado or Florida.

**Table 1:** Feline foamy virus (FFV) seroprevalence of stray domestic cats in Colorado, Southern California and Florida, broken down by sex and age, varies across and between all locations

Location	Variable	FFV seroprevalence (%)	Lower 95% CI	Upper 95% CI	Sample size (n)
Southern California	Female	77.6	65.8	86.9	67
Sourthern California	Male	73.3	63.8	81.5	105
Southern California	Young	38.7	21.9	57.8	31
Southern California	Adult	83.0	75.7	88.8	141
Colorado	Female	39.3	26.5	53.3	56
Colorado	Male	67.4	52.5	80.1	49
Colorado	Young	27.8	9.7	53.5	18
Colorado	Adult	57.5	46.4	68.0	87
Florida	Female	35.9	14.1	61.7	17
Florida	Male	50.0	23.0	77.0	14
Florida	Young	23.1	5.0	53.8	13
Florida	Adult	55.6	30.8	78.5	18
Overall	Female	57.1	48.5	65.5	140
Overall	Male	69.6	62.1	76.5	168
Overall	Young	32.3	20.9	45.3	62
Overall	Adult	72.0	65.9	77.5	246

**Figure 1:**
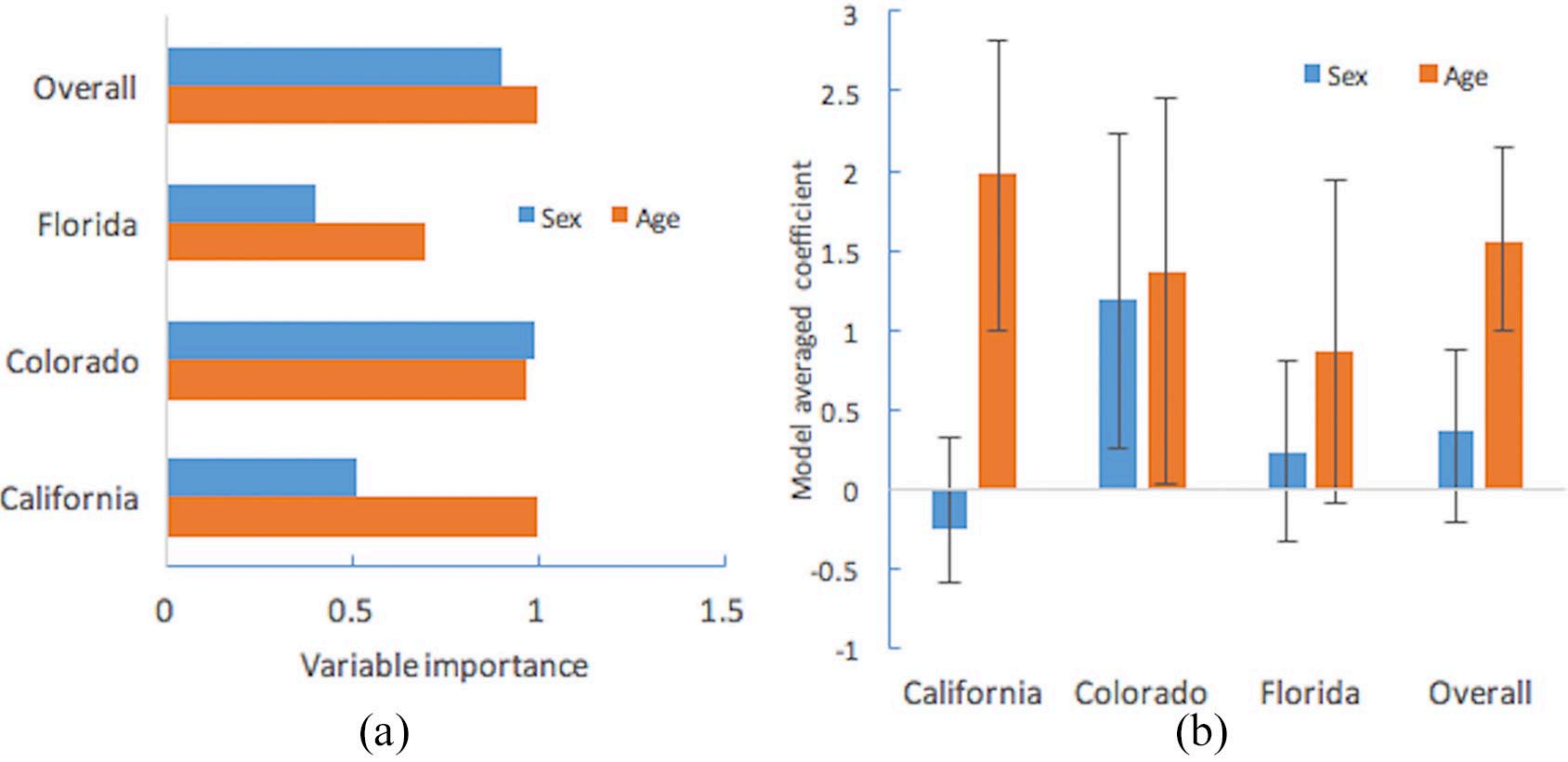
Adults are at the highest risk of feline foamy virus (FFV) infection in Southern California, Colorado and across all sampling locations (overall), while being male was also a risk factor in Colorado. (a) The plot displays variable importance weights for domestic cat sex or age as risk factors for FFV. The greater the variable importance weight, the more predictive it is for FFV infection in a given set of models. (b) The plot displays model averaged coefficients with 95% confidence intervals (CIs), with an averaged coefficient >0 being male or adult, and an averaged coefficient <0 being female or young. If the CI does not contain 0, the coefficient is considered significant. Sample sizes: adult domestic cats (n = 246), young domestic cats (n = 62), female domestic cats (n = 135), male domestic cats (n = 173)

Bayesian GLMs for pooled data and individual states found age and sex to be important predictors of FFV infection. Being an adult increased the likelihood of being seropositive for FFV in Southern California, Colorado and across all locations. In Colorado, male cats were found to be at a greater risk of FFV seropositivity than female cats (Figure 1).

## Discussion

Our estimate of FFV seroprevalence in stray domestic cats in three states falls within previously reported ranges in other countries.^2,6,18,19^ Interestingly, Southern California had the largest FFV seroprevalence across the three states, suggesting that regional geographical differences in stray domestic cat populations may influence the exposure rate. Southern California shelters are in markedly more urban landscapes than the Florida and Colorado shelters, suggesting more urban environments allow for more direct contact between stray cats and therefore enhance the spread of FFV in adult populations. Further studies on samples with other recorded demographic parameters could be conducted to determine whether population size, degree of outdoor exposure, degree of urbanization or other factors underlie this observation.

While samples were typically collected upon presentation to the shelter from individual, unrelated animals, some sample collections at trap–neuter–return sites were not from independent populations. This may have resulted in higher rates in certain sample locations than the overall regional population. However, assessment of other common domestic cat pathogens in this collection have recorded prevalence rates in accordance with ‘typical’ infection rates reported for feline pathogens,^19,26^ suggesting that our findings generally reflect FFV infection in stray cat populations in the sampling locations.

Adult stray domestic cats were more likely to be infected with FFV than young cats, supporting the hypothesis that FFV, in part, accumulates in a population through horizontal transmission.^6^ Other explanations for the results include unequal sample sizes across age, our modeling parameters and the antibody detection threshold of the ELISA. In addition, being male increased the likelihood of infection in Colorado stray domestic cats. Previous studies have reported that FFV prevalence does not vary between sexes;^6^ therefore, it is not clear if this result reflects a regional variation or spurious association. Additionally, fewer individuals were sampled over a shorter time period in Florida compared with Colorado and Southern California. Differences in this sampling strategy may have had an impact on comparative findings.

## Conclusions

This study reveals a high seroprevalence of FFV in stray domestic cat populations located in three areas of the USA, contributing to the growing documentation of FFV as an extremely common retroviral infection in domestic cats. Our results are important for contextualizing the potential consequences regarding FFV pathogenicity, considering there are still questions of FFVs’ role in domestic cat health. For example, FFV has been associated with increased FIV or FeLV viral loads during coinfection, suggesting that it may potentiate other chronic infections and/or contribute to immunodeficiency virus syndromes.^12,14,15^ Additionally, some other studies have associated FFV with chronic diseases of cats, including kidney disease and arthritis.^11,16^ Alternatively, FFV could represent a mutualistic infection, meaning that infection might have a positive benefit to the host. Mutualistic infections are thought to occur as a symbiotic relationship between pathogen and host that results from extended host:pathogen evolution, which may pertain to foamy viruses.^1,26^ Continued investigation of its clinical relationship to other infections should be conducted to assess risks of infection for domestic cat health, and develop rational guidelines for FFV surveillance.

## References

[1] Fields BN, et al Foamy viruses. In: Rethwilm A Lindemann D (eds). Fields virology. 6th ed. Philadelphia, PA: Lippincott Williams & Wilkins, 2013, p 1614.

[2] Linial ML Why aren’t foamy viruses pathogenic? Trends Microbiol 2000; 8: 284–289. 10838587 10.1016/s0966-842x(00)01763-7

[3] Saib A Non-primate foamy viruses. Curr Top Micriobiol Immunol 2003; 277: 197–211. 10.1007/978-3-642-55701-9_9 12908774

[4] Roy J Rudolph W Juretzek T, et al Feline foamy virus genome and replication strategy. J Virol 2003; 77: 11324–11331. 14557618 10.1128/JVI.77.21.11324-11331.2003 PMC229293

[5] Alke A Schwantes A Zema M, et al Characterization of the humoral immune response and virus replication in cats experimentally infected with feline foamy virus. Virology 2000; 275: 170–176. 11017797 10.1006/viro.2000.0537

[6] Winkler IG Lochelt M Flower RLP Epidemiology of feline foamy virus and feline immunodeficiency virus infections in domestic and feral cats: a seroepidemiological study. J Clin Microbiol 1999; 37: 2848–2851 10.1128/jcm.37.9.2848-2851.1999 PMC85393 10449463

[7] Liu W Lei J Liu Y, et al Feline foamy virus-based vectors: advantages of an authentic animal model. Viruses 2013; 5: 1702–1718. 23857307 10.3390/v5071702 PMC3738957

[8] Rethwilm A Foamy virus vectors: an awaited alternative to gammaretro- and lentiviral vectors. Curr Gene Ther 2007; 7: 261–271. 17969559 10.2174/156652307781369092

[9] Zemba M Alke A Bodem J, et al Construction of infectious feline foamy virus genomes: cat antisera do not cross-neutralize feline foamy virus chimera with serotype-specific Env sequences. Virology 2000; 266: 150–156. 10612669 10.1006/viro.1999.0037

[10] Ledesma-Feliciano C Hagen S Troyer R, et al Replacement of feline foamy virus bet by feline immunodeficiency virus *vif* yields replicative virus with novel vaccine candidate potential. Retrovirol 2018; 15: 38. DOI: 10.1186/s12977-018-0419-0. 10.1186/s12977-018-0419-0 PMC5956581 29769087

[11] German AC Harbour DA Helps CR, et al Is feline foamy virus really apathogenic? Vet Immunol Immunopathol 2008; 123: 114–118. 18342375 10.1016/j.vetimm.2008.01.035

[12] Cavalcante LTF Muniz CP Jia H, et al Clinical and molecular features of feline foamy virus and feline leukemia virus co-infection in naturally-infected cats. Viruses 2018; 10: 702. DOI: 10.3390/v10120702. 10.3390/v10120702 PMC6315984 30544924

[13] Yamamoto JK Hansen H Ho EW, et al Epidemiologic and clinical aspects of feline immunodeficiency virus infection in cats from the continental United States and Canada and possible mode of transmission. J Am Vet Med Assoc 1989; 194: 213–220. 2537269

[14] Zenger E Brown WC Song W, et al Evaluation of cofactor effect of feline synctium-forming virus on feline immunodeficiency virus infection. Am J Vet Res 1993; 54: 713–718. 8391229

[15] Powers JA Chiu ES Kraberger SJ, et al Feline leukemia virus disease outcomes in a domestic cat breeding colony: relationship to endogenous FeLV and other chronic viral infections. J Virol 2018; 92: e00649–18. DOI: 10.1128/JVI.000649-18. 10.1128/JVI.00649-18 PMC6146681 29976676

[16] Pedersen NC Pool RR O-Brien T Feline chronic progressive polyarthritis. Am J Vet Res 1980; 41: 522–535. 6250422

[17] Lin JA Cheng MC Inoshima Y, et al Seroepidemiological survey of feline retrovirus infections in cats in Taiwan in 1993 and 1994. J Vet Med Sci 1995; 57: 161–163. 7756412 10.1292/jvms.57.161

[18] Mochizuki M Akuzawa M Nagatomo H Serological survey of the iriomote cat (*Felis iriomotensis*) in Japan. J Wildl Dis 1990; 26: 236–245. 2110982 10.7589/0090-3558-26.2.236

[19] Bleiholder A Muhle M Hechler T, et al Pattern of seroreactivity against feline foamy virus proteins in domestic cats from Germany. Vet Immunol Immunopathol 2011; 143: 292–300. 21724269 10.1016/j.vetimm.2011.06.007

[20] Pedersen NC Feline syncytium-forming virus infection. In: Holyworth J (ed). Diseases of the cat. Philadelphia, PA: WB Saunders, 1986, pp 268–272.

[21] Carver S Bevins SN Lappin MR, et al Pathogen exposure varies widely among sympatric populations of wild and domestic felids across the United States. Ecol Appl 2016; 26: 367–381. 27209780 10.1890/15-0445

[22] Romen F Pawlita M Sehr P, et al Antibodies against Gag are diagnostic markers for feline foamy virus infections while Env and Bet reactivity is undetectable in a substantial fraction of infected cats. Virology 2006; 345: 502–508. 16297422 10.1016/j.virol.2005.10.022

[23] Benjamini Y Hochberg Y Controlling the false discovery rate: a practical and powerful approach to multiple testing. J R Stat Soc Series B 1995; 57: 289–300.

[24] Hadfield JD MCMC methods for multi-response generalized linear mixed models: the MCMCglmm R package. J Stat Soft 2010; 33: 1–22.

[25] Stutzman-Rodriguez K Rovnak J VandeWoude S Domestic cats seropositive for *Felis catus* gammaherpesvirus 1 are often qPCR negative. Virology 2016; 498: 23–30. 27540873 10.1016/j.virol.2016.07.027 PMC8366510

[26] Rethwilm A Bodem J Evolution of foamy viruses: the most ancient of all retroviruses. Viruses 2013;5: 2349–2374. 24072062 10.3390/v5102349 PMC3814592

